# Glycosylation of Fluorophenols by Plant Cell Cultures

**DOI:** 10.3390/ijms10051942

**Published:** 2009-04-27

**Authors:** Kei Shimoda, Naoji Kubota, Yoko Kondo, Daisuke Sato, Hiroki Hamada

**Affiliations:** 1 Department of Chemistry, Faculty of Medicine, Oita University, 1-1 Hasama-machi, Oita 879-5593, Japan; 2 Department of Life Science, Okayama University of Science, 1-1 Ridai-cho, Okayama 700-0005, Japan

**Keywords:** Fluorophenol, Glycosylation, Nicotiana Tabacum, Plant Cell Cultures

## Abstract

Fluoroaromatic compounds are used as agrochemicals and released into environment as pollutants. Glycosylation of 2-, 3-, and 4-fluorophenols using plant cell cultures of *Nicotiana tabacum* was investigated to elucidate their potential to metabolize these compounds. Cultured *N. tabacum* cells converted 2-fluorophenol into its β-glucoside (60%) and β-gentiobioside (10%). 4-Fluorophenol was also glycosylated to its β-glucoside (32%) and β-gentiobioside (6%) by *N. tabacum* cells. On the other hand, *N. tabacum* glycosylated 3-fluorophenol to β-glucoside (17%).

## 1. Introduction

Halophenolic compounds are used to agrochemicals and pharmaceuticals, and may cause serious environmental contamination [1]. For example, the number of fluorine-containing agricultural has increased in number faster than non-fluorinated agrochemicals over the past 15 years, going from 4% to approximately 9% of all agrochemicals [1]. These compounds are primarily used as herbicides (48%), insecticides (23%), and fungicides (18%). There have been many reports on biological degradation of halophenols by microorganisms [1–9], but halophenols containing stable carbon-halogen bonds in their structures have been described to be much more resistant to microbial degradation than otherwise substituted analogs [1].

Plant cell cultures are ideal systems for studying biotransformations specific to plants such as glycosylation reactions [10]. Plant cells can convert exogenous toxic compounds into glycosides [11–15], so glycosylation of halophenols by plant cell cultures is of importance from the viewpoint of pollution control. This study focused on the metabolism of monofluorophenols by plant cell cultures of *Nicotiana tabacum*.

## 2. Results and Discussion

### 2.1. Glycosylation of 2-fluorophenol (**1**)

**Scheme 1.**
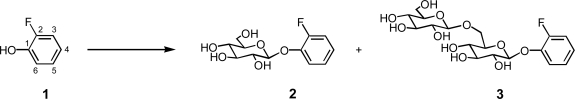
Glycosylation of 2-fluorophenol (**1**) to 2-fluorolphenyl β-glucoside (**2**) and 2-fluorolphenyl β-gentiobioside (**3**) by *N. tabacum* cell cultures.

After plant cell cultures of *N. tabacum* were incubated with 2-fluorophenol (**1**) for five days, the biotransformation products **2** and **3** (Scheme 1) were isolated from the cells by homogenization and extraction with methanol. On the other hand, no products were detected in the medium by HPLC analysis. No additional glycosylation products were detected in the methanol extracts of the cells despite careful HPLC analyses. HPLC analyses of the control cell cultures of *N. tabacum*, which had not been treated with 2-, 3-, and 4-fluorophenols (**1**, **4**, and **6**), showed that there were no mono-fluorophenols and their glycosides in both medium and *N. tabacum* cells. On the basis of the spectroscopic analyses such as FABMS, ^1^H- and ^13^C-NMR, the chemical structures of the products were determined to be 2-fluorophenyl β-glucoside (**2**, 60%) [16] and 2-fluorophenyl β-gentiobioside (**3**, 10%) of which **3** has not been identified before.

The FABMS spectrum of **3** included a pseudomolecular ion [M+Na]^+^ peak at *m*/*z* 459, indicating that the compound was composed of one molecule of the substrate **1** and two hexoses. The ^1^H-NMR spectrum of **3** showed two anomeric proton signals at *δ* 4.36 (1H, *d*, *J*=7.6 Hz) and 4.96 (1H, *d*, *J*=7.6 Hz). From the coupling pattern of the proton signals and the chemical shifts of the carbon signals due to the sugar moiety of **3**, it was indicated to be β-gentiobioside [17]. Additionally, HMBC correlations were observed between the anomeric proton signal at *δ* 4.96 (H-1′) and the carbon signal at *δ* 146.3 (C-1) and between the anomeric proton signal at *δ* 4.36 (H-1″) and the carbon signal at *δ* 69.6 (C-6′) which confirms that the inner glucopyranosyl residue was attached to the phenolic hydroxyl group of 2-fluorophenol (**1**) and that the pair of β-d-glucopyranosyl residues was 1,6-linked. Thus, the structure of **3** was determined to be 2-fluorophenyl β-gentiobioside.

**Figure 1.**
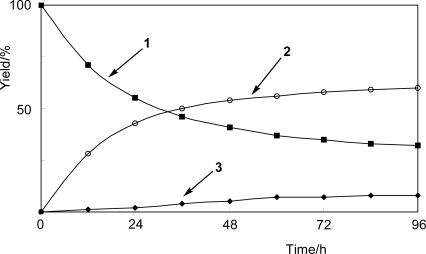
Time-course of glycosylation of 2-fluorophenol (**1**) to its β-glucoside **2** and β-gentiobioside **3** by *N. tabacum* cell cultures.

A time-course experiment was carried out to investigate the scope of the ability of *N. tabacum* cell cultures to glycosylate 2-fluorophenol (**1**) (Figure 1). The amount of products **2** and **3** increased with incubation time. In our recent papers, we reported that the amount of disaccharides such as β-primeverosides and β-vicianosides increased with a corresponding decrease of that of β-glucosides in the biotransformation of phenolic compounds, i.e., capsaicinoids, by plant cell cultures and that conversion of the substrates to their β-glucosides occurred first, followed by further glycosylation to the corresponding disaccharides [18]. However, it is not clear that stepwise glycosylations occurred in the present case.

Recently, we reported that cultured *Eucalyptus perriniana* cells converted 2-, 3-, and 4-fluorophenols into the corresponding β-glucosides [16]. On the other hand, it has been reported that exogenously added α-tocopherol was glycosylated to its β-gentiobioside by *N. tabacum* cell cultures [17]. The glycosylation of exogenous phenolic compounds to their gentiobiosides was a characteristic reaction for *N. tabacum* cell cultures.

### 2.2. Glycosylation of 3-fluorophenol (**4**)

**Scheme 2.**
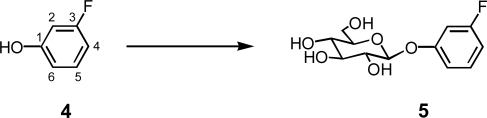
Glycosylation of 3-fluorophenol (**4**) to 3-fluorophenyl β-glucoside (**5**) by *N. tabacum* cell cultures.

The substrate 3-fluorophenol (**4**), was subjected to biotransformation with the same transformation system as used with 2-fluorophenol (**1**) (Scheme 2). Product **5** (17%) was detected in the methanol cell extracts. The structure of product **5** was determined to be 3-fluorophenyl β-glucoside by spectroscopic methods [16]. No further glycosylation products such as β-gentiobioside were obtained.

**Figure 2.**
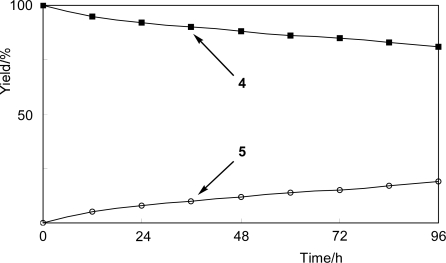
Time-course of glycosylation of 3-fluorophenol (**4**) to its β-glucoside **5** by *N. tabacum* cell cultures.

The time-course of the biotransformation of 3-fluorophenol (**4**) showed that the amount of product **5** increased with time during the reaction with *N. tabacum* cell cultures (Figure 2).

### 2.3. Glycosylation of 4-fluorophenol (**6**)

**Figure 3.**
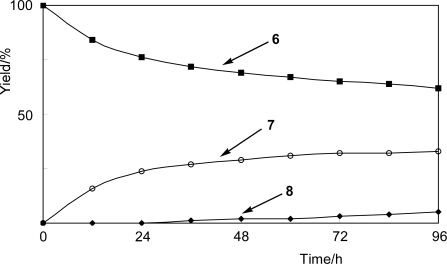
Time-course of glycosylation of 4-fluorophenol (**6**) to its β-glucoside **7** and β-gentiobioside **8** by *N. tabacum* cell cultures.

**Scheme 3.**
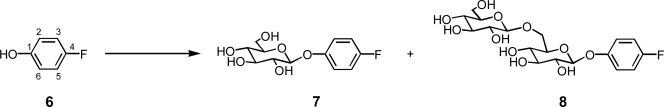
Glycosylation of 4-fluorophenol (**6**) to 4-fluorolphenyl β-glucoside (**7**) and 4-fluorolphenyl β-gentiobioside (**8**) by *N. tabacum* cell cultures.

Incubation of *N. tabacum* cell cultures with 4-fluorophenol (**6**) for five days gave the biotransformation products **7** and **8** (Scheme 3). The structures of **7** and **8** were determined as 4-fluorophenyl β-glucoside (32%) [16] and 4-fluorophenyl β-gentiobioside (6%), respectively. The β-gentiobioside **8** was identified for the first time. The time-course in the biotransformation of **6** showed that the amounts of β-glucoside **7** and β-gentiobioside **8** increased with time (Figure 3).

The FABMS spectrum of **8** included an ion attributed to [M+Na]^+^ pseudomolecular ion (*m*/*z* 459), suggesting that **8** contained one substrate molecule **6** and two hexoses. Its ^1^H- and ^13^C-NMR data were in good agreement with those of β-gentiobioside **3**. The HMBC spectrum of **8** included correlations between the anomeric proton signal at *δ* 4.83 (H-1′) and the carbon signal at *δ* 155.1 (C-1) and between the anomeric proton signal at *δ* 4.38 (H-1″) and the carbon signal at *δ* 69.7 (C-6′), indicating that the inner glucopyranosyl residue was attached to the phenolic hydroxyl group of 4-fluorophenol (**6**) and that the pair of β-d-glucopyranosyl residues was 1,6-linked. The structure of **8** was determined to be 4-fluorophenyl β-gentiobioside.

## 3. Conclusions

Halophenols, i.e., 2-, 3-, and 4-fluorophenols, exogenously added to *N. tabacum* cell cultures were efficiently transformed to their corresponding glucoconjugates, which are accumulated in the cells. The substrates 2- and 4-fluorophenol were converted into the corresponding β-glucosides and β-gentiobiosides, while 3-fluorophenol was glycosylated to only a β-glucoside.

Plant cells can conjugate sugar residues, not only to endogenous metabolic intermediates but also to xenobiotics [19]. Glycosylation reactions serve diverse functions in plants, i.e., increasing the solubility and stability of the aglycones, decreasing the toxicity of xenobiotics, and activating biosynthetic intermediates. The present biotransformation system using *N. tabacum* cell cultures would be useful for glycosylation of fluoroaromatic compounds. Furthermore, we have reported that immobilized plant cells of *N. tabacum* in sodium alginate gel glycosylated bisphenol A and benzophenone in higher yields than normal *N. tabacum* cell cultures [20], so immobilized *N. tabacum* cells could be applicable for phytoremediation of fluorophenols to be removed from contaminated water. The glycosylation of these compounds by immobilized *N. tabacum* cells will be reported in the near future.

## 4. Experimental Section

### 4.1. General

The substrates, 2-, 3-, and 4-fluorophenols, were purchased from Sigma-Aldrich Co. The NMR spectra were recorded in CD_3_OD using a Varian XL-400 spectrometer (^1^H at 400 MHz and ^13^C at 100 MHz, respectively). The chemical shifts were expressed in δ (ppm) referring to tetramethylsilane. The FABMS spectra were measured using a JEOL MStation JMS-700 spectrometer. HPLC was carried out on a CrestPak C18S column (150 x 4.6 mm) [solvent: dioxane:H_2_O (1:9, v/v); detection: UV (270 nm); flow rate: 1.0 mL/min]. Cultured suspension cells of *N. tabacum* were cultivated in 300 mL conical flasks containing Murashige and Skoog’s medium (100 mL, pH 5.7) and grown with continuous shaking on a rotary shaker (120 rpm) at 25 °C in the dark.

### 4.2. Biotransformation of **1**, **4**, and **6** with plant cell cultures of N. tabacum

Biotransformation experiments were performed by addition of substrate (0.1 mmol/flask) into ten 300 mL conical flasks containing the suspension of cultured cells of *N. tabacum* and the cultures were incubated at 25°C for five days on a rotary shaker (120 rpm) in the dark. After incubation, the cells and medium were separated by filtration with suction. The filtered medium was extracted with ethylacetate. The medium was further extracted with *n*-butanol. The cells were homogenized and extracted with methanol. The glycoside products were detected in methanol fractions by HPLC analyses. The methanol fraction was concentrated and partitioned between water and ethylacetate. The ethylacetate fractions were combined and analyzed by the HPLC. The water fraction was applied to a Diaion HP-20 column and the column was washed with water followed by elution with methanol. The methanol eluate was subjected to preparative HPLC to give glycoside products.

Spectral data of new compounds are as follows.

#### 2-Fluorophenyl β-gentiobioside (**3**):

FABMS *m/z*: 459 [M+Na]^+^; ^1^H-NMR δ: 3.17–3.85 (11H, m, H-2′, 2″, 3′, 3″, 4′, 4″, 5′, 5″, 6a′, 6″), 4.15 (1H, dd, *J*=12.0, 2.0 Hz, H-6b′), 4.36 (1H, d, *J*=7.6 Hz, H-1″), 4.96 (1H, d, *J*=7.6 Hz, H-1′), 6.96–7.01 (1H, m, H-4), 7.06–7.13 (2H, m, H-3, H-6), 7.34 (1H, td, *J*=8.0, 1.2Hz, H-5); ^13^C-NMR δ: 62.6 (C-6″), 69.6 (C-6′), 71.2 (C-4″), 71.5 (C-4′), 74.7 (C-2′), 75.0 (C-2″), 77.4, 77.8 (C-3′, 3″, 5′, 5″), 102.4 (C-1′), 104.6 (C-1″), 116.9 (1C, d, *J* _F-3C_ =18 Hz, C-3), 119.3 (C-6), 123.8 (C-4), 125.6 (C-5), 146.3 (1C, d, *J* _F-1C_ =11 Hz, C-1), 153.9 (1C, d, *J* _F-2C_ =244 Hz, C-2).

#### 4-Fluorophenyl β-gentiobioside (**8**):

FABMS *m/z*: 459 [M+Na]^+^; ^1^H-NMR δ: 3.20–3.91 (11H, m, H-2′, 2″, 3′, 3″, 4′, 4″, 5′, 5″, 6a′, 6″), 4.17 (1H, dd, *J*=12.4, 2.0 Hz, H-6b′), 4.38 (1H, d, *J*=8.0 Hz, H-1″), 4.83 (1H, d, *J*=8.0 Hz, H-1′), 6.98–7.02 (2H, m, H-3, 5), 7.12–7.15 (2H, m, H-2, H-6); ^13^C-NMR δ: 62.6 (C-6″), 69.7 (C-6′), 71.3 (C-4″), 71.5 (C-4′), 74.7 (C-2′), 75.1 (C-2″), 77.3, 77.7, 77.9 (C-3′, 3″, 5′, 5″), 102.7 (C-1′), 104.6 (C-1″), 116.5 (2C, d, *J* _F-3C, 5C_ =23 Hz, C-3, C-5), 119.1 (C-2, C-6), 155.1 (C-1), 159.3 (1C, d, *J* _F-4C_ =245 Hz, C-4).

### 4.3. Time-course experiment

Suspension cells of *N. tabacum* (50 g, fr. wt) was partitioned into each of eight 300 mL conical flasks containing Murashige and Skoog’s medium (100 mL). Substrate (0.1 mmol) dissolved in ethanol was administered to each of conical flasks and the mixtures were incubated on a rotary shaker at 25 °C. At 12 h intervals, one of the flasks was taken out from the rotary shaker, and the cells and medium were separated by filtration. The extraction and analysis procedures were same as described in Section 3.2. The yield of the products was determined on the basis of the peak area from HPLC and expressed as a relative percentage to the total amount of the whole reaction products extracted.
